# Associations between Socio-Demographic Factors and Hypertension Management during the COVID-19 Pandemic: Preliminary Findings from Malaysia

**DOI:** 10.3390/ijerph18179306

**Published:** 2021-09-03

**Authors:** Mohamed Hassan Elnaem, Nur Hasyimah Kamarudin, Nabeel Kashan Syed, Hasniza Zaman Huri, Inderpal Singh Dehele, Ejaz Cheema

**Affiliations:** 1 Department of Pharmacy Practice, Faculty of Pharmacy, International Islamic University Malaysia, Kuantan 25710, Malaysia; shima.shi98@gmail.com; 2 Quality Use of Medicines Research Group, Faculty of Pharmacy, International Islamic University Malaysia, Kuantan 25710, Malaysia; 3 Pharmacy Practice Research Unit, Department of Clinical Pharmacy, Faculty of Pharmacy, Jazan University, Jizan 45142, Saudi Arabia; nabeelsyed2020@outlook.com; 4 Department of Clinical Pharmacy and Pharmacy Practice, Faculty of Pharmacy, Universiti Malaya, Kuala Lumpur 50603, Malaysia; hasnizazh@um.edu.my; 5 School of Pharmacy, University of Birmingham, Birmingham B15 2TT, UK; I.S.Dehele@bham.ac.uk; 6 School of Pharmacy, University of Management and Technology, Lahore 54770, Pakistan; cheemaejaz@hotmail.com

**Keywords:** hypertension, pandemic, perspectives, blood pressure control, health literacy, adherence, COVID-19 pandemic, Malaysia

## Abstract

The perspectives of hypertensive patients on the state of hypertension control during the ongoing pandemic restrictions have not been extensively studied in Malaysia. Therefore, this study aimed to assess the impact of socio-demographic factors, health literacy, and adherence on the overall hypertension management in a group of Malaysian hypertensive patients during the COVID-19 pandemic. An anonymous, online cross-sectional study was conducted over three months that involved a group of Malaysian adults with hypertension. A validated, self-administered 30-item questionnaire was prepared in Malay and English languages on Google Forms. The link was then distributed to participants on social media (Facebook and WhatsApp). Following survey validation, a pilot study with 30 participants who met the inclusion criteria was carried out. The total scores for health literacy, adherence, and pandemic impact on hypertension control were calculated and compared across all independent variables. In a total of 144 study participants, controlled blood pressure was reported in 77% (N = 111). There were good levels of adherence and health literacy scores but moderate levels of pandemic impact scores. The total adherence scores showed a statistically significant difference between age groups (χ^2^ = 6.48, *p* = 0.039) and those who reported having controlled and uncontrolled blood pressure (U = 1116, *p* = 0.001). Moreover, the analysis revealed statistically significant differences in total pandemic impact scores based on the age group (χ^2^ = 15.008, *p* = 0.001), household income (χ^2^ = 6.887, *p* = 0.032), employment (U = 1712, *p* = 0.006), and marital status (U = 520.5, *p* < 0.001). The youngest age group (18–39) years, the lowest income group, unemployed and unmarried individuals, had significantly higher pandemic impact scores. This denotes that those individuals were more prone to be negatively affected by the pandemic regarding their hypertension management. Most participants reported relatively controlled blood pressure and good levels of health literacy as well as adherence amidst the pandemic. To a moderate extent, study participants perceived that the pandemic had a negative effect on hypertension management. The perceived negative impact of the pandemic was attributed to several socio-demographic factors, such as age, household income, employment, and marital status.

## 1. Introduction

Hypertension is one of the most prevalent non-communicable diseases (NCDs), with a global prevalence of 20% to 25%, according to WHO 2015 key facts sheets [1]. Due to its asymptomatic nature, hypertension or high blood pressure is often referred to as a silent killer disease [2]. Maintaining proper blood pressure (BP) control is challenging, with a reported success rate of only 40%, although a high proportion of patients receive treatment [3]. Global data shows that less than one in five patients manage to maintain proper BP control [1]. In Malaysia, hypertension is a prevalent healthcare issue where three among every ten individuals have hypertension [4]. Moreover, the analysis of the COVID-19 pandemic data showed that a large proportion of hospitalised COVID-19 patients also had hypertension [5]. Those hypertensive patients had poor prognosis with significant need for mechanical ventilation and higher mortality risk [6].

Health literacy and medication adherence are two main challenges for controlling hypertension in clinical settings [7,8,9]. Health literacy affects a patient’s level of self-care, medication knowledge, and adherence [9]. According to the American Heart Association, health literacy can help patients improve their health status, better understand disease processes, and lower their risk of hospitalisation [10]. Additionally, the silent nature of the disease complicates medication adherence, as non-adherence may not immediately manifest as unpleasant symptoms [11]. Thus, adherence has been highlighted as a critical issue in hypertension management, with poor adherence identified as a key contributor to uncontrolled hypertension [12]. This should be a major concern for the healthcare systems, as hypertensive patients with poorly controlled blood pressure are at an increased risk of developing or worsening pre-existing cardiovascular disease (CVD), cerebrovascular, and renal diseases [3].

Globally, most sectors, including healthcare, have been negatively impacted by the COVID-19 pandemic [13]. According to the World Health Organization (WHO) rapid assessment of the impact of COVID-19 pandemic on NCD resources, approximately 75% of participating countries reported disruptions to NCD services, including hypertension management [14]. Healthcare services for chronic disease patients were at risk of disruptions during the ongoing pandemic. Hypertension, as is the case with other NCDs, requires regular and long-term care [14]. These disruptions must be acknowledged and addressed by the health care providers to help patients with NCD maintain proper control of their conditions [15]. The Hypertension Cardiovascular Outcomes Prevention Evidence in Asia (HOPE Asia) acknowledged that management of hypertension might be affected by the pandemic-related restrictions and thus recommended telemedicine strategies to be implemented [16].

However, the perspectives of hypertensive patients on the state of blood pressure control during the current pandemic restrictions need further investigations. In addition, further information on the important demographic data to be considered in the context of pandemic impact on disease management is still required. Therefore, this study aimed to assess the impact of socio-demographic factors, health literacy, and adherence on the overall hypertension management in a group of Malaysian hypertensive patients during the COVID-19 pandemic.

## 2. Materials and Methods

### 2.1. Study Design

This descriptive anonymous, online, cross-sectional study involving a sample of adults with hypertension in Malaysia was conducted for a period of three months between January and April 2021. The study used a validated, self-administered survey prepared in Malay and English languages on Google Forms and was disseminated on social media. Participants were asked to answer only one of the versions to avoid duplicate responses. 

### 2.2. Ethical Approval

Ethical approval for this study was provided by IIUM Research Ethics Committee (IREC 2020-176). The participation information sheet and informed consent were included in the online survey form. Participants were briefed on the strict confidentiality of their information and the anonymous use of their data for scientific research purposes only. By approving the consent form, participants were deemed to have consented to participate in this research. They were also given the freedom to withdraw their consent at any time during the study.

### 2.3. Inclusion and Exclusion Criteria 

Adults aged 18 years old and above with hypertension were eligible to be included in this study. The participants were eligible for inclusion if they had received a diagnosis of hypertension with or without comorbidity before the declaration of the COVID-19 outbreak as a pandemic by the WHO on 11 March 2020 [17]. Pregnant patients were excluded from the study.

### 2.4. Sample Size

Using the Raosoft sample size calculator, assuming an estimated proportion of 72% based on the most recent estimate from a similar setting [14] and a 95% confidence interval, and upon confirmation that at least 260 potential respondents had received the survey form, the minimum required sample size was estimated to be 142.

### 2.5. Instrument Structure and Translation

A self-developed, pre-validated 30-item questionnaire was prepared in Malay and English languages on Google Forms, and the link was then distributed to participants on social media (Facebook and WhatsApp). 

The questionnaire comprised of four parts:

- Part 1 (nine items to cover the general socio-demographic details and the average BP reading).
- Part 2 (five statements to assess their health literacy level).
- Part 3 (eight statements to assess their adherence level). This part was developed with permission from the original developer by adopting and adapting Hill–Bone HBP compliance to the high blood pressure therapy scale (HB-HBP) [18].
- Part 4 (eight statements to assess the impact of the pandemic on hypertension control).

The translation from English to Malay was carried out following the forward-backward translation method. These three versions, including the original version, were then compared to ensure consistency before proceeding to the pilot study.

### 2.6. Validity Testing

Five experts in pharmacy practice evaluated the questionnaire’s content validity by estimating the content validity index for each item (I-CVI) to ascertain its relevance and clarity. The I-CVI should be at least 0.78 with a minimum of three experts [19]. Any item with I-CVI < 0.78 for relevance was discarded from the questionnaire, while any item with I-CVI < 0.78 was improved for better clarity based on the experts’ suggestions. For example, an item asked about all the diet recommendations in one statement in the health literacy section. Based on the expert’s suggestion, the item was revised to be more concise and focused only on salt restriction. Item 5 in the health literacy section was also rephrased accordingly as per the expert’s recommendation. In the first draft, this item did not precisely refer to the duration of the disease or its treatment.

### 2.7. Pilot Study and Reliability Testing

Upon validation, a pilot study was conducted on 30 participants who fulfilled the inclusion criteria. The data obtained from the participants in the pilot study were excluded from the main data analysis. The questionnaire was then tested for its reliability and internal consistency. The values of Cronbach’s alpha for each part, excluding the first part (Part 1: Socio-demographic details), were calculated to determine the internal consistency. Some items were omitted to obtain Cronbach’s alpha values within an acceptable range. The final values of Cronbach’s alpha for Part 2 (health literacy level assessment), Part 3 (adherence level assessment), and Part 4 (the impact of the pandemic on hypertension control) are 0.6, 0.9, and 0.7, respectively. These obtained values excluded the presence of poor or unacceptable items [20]. Therefore, the questionnaire was then finalised and disseminated for the main data collection.

### 2.8. Data Collection

The final survey was distributed through online media, mainly social media platforms (Facebook and WhatsApp), using the convenience sampling method. The online medium was used to disseminate the survey form to avoid the additional risk of face-to-face interaction during the current COVID-19 restrictions. A minimum of three weekly reminders were sent throughout the twelve weeks dedicated for data collection. The total scores of health literacy, adherence, and pandemic impact were calculated for each participant. Higher scores for health literacy (total = 5) and adherence (total = 24) are direct measures of their levels, while higher scores of the pandemic impact (total = 24) denote greater negative impact of the pandemic on blood pressure control from patients’ perspectives.

### 2.9. Statistical Analysis

Data were analysed using statistical package for the social sciences (SPSS-10 Inc., Chicago, IL, USA) version 22.0. The responses were analysed using descriptive statistics in the form of frequencies and percentages. The total scores for three of the main variables: health literacy, adherence, and impact of the pandemic of each participant, were calculated. The association between the categorical variables was examined using chi-square test. The significance of the differences between patients’ demographics, blood pressure control as categorical variables, and the total scores of all three main continuous variables were examined using Mann–Whitney and Kruskal–Wallis tests, considering they did not meet the assumption of normal distribution [21,22]. A *p*-value of <0.05 was set as the significance level for all comparisons.

## 3. Results

### 3.1. Demographics of Study Participants and Their Blood Pressure Control

A total of 144 patients consented to participate in the study. About 68.8% of respondents were 40–59 years old, and 51.4% were female. Moreover, 44.4% of respondents were in the household income group of M40, meaning their income was between RM 4850 to 10,959. Participants were categorised into two main groups, controlled and uncontrolled BP, referring to their average measured BP over the last seven days. Controlled blood pressure was reported in 77% (N = 111). Frequencies and percentages of participants’ demographic details are summarised in Table 1.

### 3.2. Health Literacy

#### 3.2.1. Health Literacy (Overall Responses)

The total score for the health literacy part that consisted of five items was five. Total health literacy score median = 4 and interquartile range (IQR) = 2. Higher scores were regarded as indicators of good health literacy. Table 2 summarises the overall responses to health literacy items presented in frequencies and percentages.

#### 3.2.2. Health Literacy (Inferential Statistics)

A chi-square test of independence was conducted between patients’ demographics and health literacy items. All expected cell frequencies were greater than five. There was a statistically significant association between gender and patient’s literacy that hypertension treatment is lifelong (χ^2^ (1) = 4.49, *p* = 0.03). There was also a statistically significant association between monthly household income and the patient’s literacy; hypertension cannot be cured in few weeks after initiating treatment (χ^2^ (2) = 6.32, *p* = 0.04).

### 3.3. Adherence

#### 3.3.1. Adherence (Overall Responses)

The total score for the adherence part that consisted of eight items was 24. Total adherence score median = 21 and IQR = 3. Higher scores were regarded as indicators of good adherence. Table 3 summarises the overall responses to adherence items presented in frequencies and percentages.

#### 3.3.2. Adherence (Inferential Statistics)

A Kruskal–Wallis H test was run to determine if there were differences in total adherence scores between the three age groups: “18–39 years”, “40–59 years”, and “60 years and above”. Distributions of total adherence scores were not similar for all groups, as assessed by visual inspection of a boxplot. The distributions of total adherence scores were statistically significantly different between age groups (χ^2^ (2) = 6.48, *p* = 0.039). Subsequently, pairwise comparisons were performed using Dunn’s (1964) procedure with a Bonferroni correction for multiple comparisons. Adjusted *p*-values are presented. Values are mean ranks unless otherwise stated. This post hoc analysis revealed statistically significant differences in total adherence scores between the “60 years and above” group (95.42) and “40–59 years” group (69.99) (*p* = 0.049), but not between any other group combination.

A Mann–Whitney U test was run to determine differences in total adherence scores between those who reported controlled and uncontrolled blood pressure. Distributions of the total adherence scores were not similar, as assessed by visual inspection. Total adherence scores for those who reported controlled blood pressure (mean rank = 78.95) were statistically significantly higher than for those reported uncontrolled blood pressure (mean rank = 50.82) (U = 1116, z = −3.434, *p* = 0.001).

### 3.4. Impact of the COVID-19 Pandemic

#### 3.4.1. Pandemic Impact (Overall Response)

The total score for the pandemic impact on hypertension control that consisted of eight items was 24. Total pandemic impact scores median = 12 and IQR = 4.8. Higher scores were regarded as indicators of the negative impact of the pandemic on hypertension control. Table 4 summarises the overall responses to pandemic impact items presented in frequencies and percentages.

#### 3.4.2. Pandemic Impact (Inferential Statistics)

A chi-square test of independence was conducted between patients’ demographics and pandemic impact items. All expected cell frequencies were greater than five. There was a statistically significant association between gender and perception that hypertension treatment became more complex during the pandemic than before (χ^2^ (2) = 5.93, *p* = 0.05). Also, there was a statistically significant association between level of education and the individual’s perspective on more reliance on self-monitoring of BP during the pandemic (χ^2^ (2) = 6.41, *p* = 0.04).

A Kruskal–Wallis H test was conducted to determine if there were differences in total pandemic impact scores between the three age groups: “18–39 years”, “40–59 years”, and “60 years and above” and three household income groups: “B40 (income less than 4.850 RM)”, “M40 (income in the range of 4.850–10.959 RM)”, and “T20 (income above 10.959 RM)”. Distributions of total pandemic impact scores were not similar for all groups, as assessed by visual inspection of a boxplot. The distributions of pandemic impact scores had statistically significant differences between age groups (χ^2^ (2) = 15.008, *p* = 0.001) and between household income groups (χ^2^ (2) = 6.887, *p* = 0.032). Subsequently, pairwise comparisons were performed using Dunn’s (1964) procedure with a Bonferroni correction for multiple comparisons. Adjusted *p*-values are presented. Values are mean ranks unless otherwise stated. This post hoc analysis revealed statistically significant differences in total pandemic impact scores between the “18–39 years” group (100.33) and both groups of “60 years and above” (66.03) (*p* = 0.019) and “40–59 years” (66.09) (*p* < 0.001). Furthermore, it revealed statistically significant differences in total pandemic impact scores between the “B40” group (84.37) and the “M40” group (65.66) (*p* = 0.045), but not between any other group combination.

A Mann–Whitney U test was conducted to determine differences in total pandemic impact scores between the employed and unemployed participants and between the married and unmarried participants. Distributions of the total pandemic impact scores were not similar, as assessed by visual inspection. Total pandemic impact scores for the unemployed participants (mean rank = 85.42) were statistically significantly higher than for those employed individuals (mean rank = 65.41) (U = 1712, z = −2.772, *p* = 0.006). In addition, total pandemic impact scores for those unmarried individuals (mean rank = 109.21) were statistically significantly higher than for those married individuals (mean rank = 66.23) (U = 520.5, z = −4.395, *p* < 0.001).

## 4. Discussion

To the best of our knowledge, this is one of the first studies that has investigated the impact of the COVID-19 pandemic on BP control among a sample of hypertensive patients in Malaysia. This study aimed to assess the impact of socio-demographic factors, health literacy, and adherence on the overall hypertension management during the COVID-19 pandemic. We also aimed to provide further insights on the patients’ perspectives about the impact of the pandemic on their hypertension disease management. The findings showed that a total of 77% (N = 111) participants had their BP under control. Our findings contrast to a previous study that included Asian patients and reported 62.4% of their study subjects to have uncontrolled BP [21]. Overall, most participants had good levels of health literacy and adherence. However, overall responses did not support a significant impact of the pandemic on hypertension control where about 77% of patients attained their target BP; there were many significant barriers to optimal hypertension disease management imposed by the pandemic.

### 4.1. Health Literacy

The overall reported level of health literacy among study participants was good, with a median score of four out of five. In a recent cross-sectional study conducted in Iran in 2020 among 210 patients, the findings showed that adequate health literacy was significantly associated with hypertension control [23]. Similarly, a study conducted in Uzbekistan in 2014 involving 209 hypertensive patients also reported a significant association between good hypertension knowledge and controlled BP [24]. On the other hand, a Turkish cross-sectional study in 2016 that involved 485 hypertensive patients in determining the effect of hypertension knowledge on blood pressure control found no significant correlation between the level of knowledge and the ratio of subjects with controlled blood pressure [25]. Furthermore, a study investigating the role of health literacy in hypertension control suggested that health information should be considered a critical part of hypertension management [23].

### 4.2. Health Literacy and Socio-Demographics

Moreover, our study findings demonstrated a statistically significant association between gender and patients’ literacy that hypertension treatment is lifelong (χ^2^ = 4.49, *p* = 0.03). Male respondents were more aware of the lifelong nature of hypertension compared to female participants. Consistently, a 2017 Chinese study that investigated the association between health literacy and hypertension management reported that more males were in the high health literacy group [9]. This point could be relevant in designing and implementing health awareness campaigns focusing on enhancing females’ awareness.

Furthermore, our findings reported a statistically significant association between monthly household income and patients’ literacy that hypertension cannot be cured in a few weeks after initiating treatment (χ^2^ = 6.32, *p* = 0.04). Participants in the highest monthly income group were the most knowledgeable that hypertension is a chronic condition compared to lower-income groups. Considering patients’ economic status and how it affects their awareness and ability to maintain proper disease management is critical, especially during the pandemic period, which has resulted in several economic challenges to individuals and the health care system [26].

### 4.3. Adherence

The overall level of adherence among study participants was good, with an average score of 20.5 out of 24. Numerous studies, including two conducted locally, also reported high adherence among most hypertensive participants [27,28,29,30,31,32,33]. In 2013, the COMFORT study that included 203 hypertensive Japanese patients reported the highest level of adherence, with 90.6% reporting an adherence rate of 90–100% [29]. Our study incorporated three elements of adherence in the questionnaire: medications, diet, and follow-up appointments to measure patients’ adherence to the overall treatment plan and how it affected their BP control. We found a significant association between total adherence scores and BP control (U = 1116, *p* = 0.001). This finding is consistent with the findings from several other studies [27,32,33,34,35]. For example, an Ethiopian study conducted by Animut et al. in 2018 reported that patients who were highly adherent to their antihypertensive medications were two times more likely to have controlled BP than those with low adherence [32]. Apart from that, a Nigerian study in 2018 measured treatment adherence to pharmacological and non-pharmacological therapies on 605 hypertensive patients. The findings demonstrated a significant association between treatment adherence (medications, smoking cessation, and exercise) with BP control, particularly baseline systolic BP [36].

### 4.4. Adherence & Socio-Demographics

Several factors may influence patients’ adherence. In our findings, the age group was the only demographic data that showed a significant difference in the total adherence scores. The older age group (60 or more) obtained higher scores than the younger group of 40–59 years. It seemed that the older population was more concerned about their medication adherence during the pandemic, considering a recent report in 2020 published by researchers in Iran and the UK that discussed the role of fear of COVID-19 on preventive behaviours [37]. It may also be attributed to the extra care given to this vulnerable population during this challenging time to avoid the undesired consequences, recognising forgetfulness as one of the top barriers to medication adherence [35,36]. Previous research from Malaysia in 2020 reported that a longer duration of hypertension, medication side effects, and use of traditional medicine were among the predictors of medication non-adherence among hypertension patients [31]. These factors should be addressed by healthcare providers and patients to optimise medication adherence and clinical outcomes [38].

### 4.5. Pandemic Impact

In measuring the impact of the pandemic on hypertension from patients’ perspectives, we incorporated essential aspects concerning the pandemic and BP control in the questionnaire. These aspects included worsening BP level, increased reliance on self-monitoring, the challenge of getting medication supplies due to COVID-19 infection risk, changes in the treatment plan, adherence to healthy diet and medications, and life-stresses. The total pandemic impact score median of 12 on a scale of 24, suggesting that there was an impact, but this negative impact was not enormous across all items. Patients in our study seem to face challenges with the pandemic related-lockdowns and COVID-19-related movement restrictions to ensure adequate medication supplies. Moreover, they had been exposed to increased life stressors in the pandemic time. In addition, they had found themselves enforced to be more dependent on self-monitoring as compared to the time prior to the pandemic. A recent report from Malaysia discussed the challenges with proceeding with optimal care for chronic conditions during the pandemic and reported concerns on medication supply and self-monitoring needs as predicted challenges in this time [15].

### 4.6. Pandemic Impact and Socio-Demographics

Importantly, our findings provided insights into the characteristics of patients who were more likely to be negatively affected by the pandemic while maintaining their hypertension control. We found a statistically significant association between gender and the perception that hypertension treatment became more complex during the pandemic than before. Females were more likely to disagree with the increased complexity of hypertension treatment than males. Concerning the gender impact on BP control, several studies reported that male patients were more prone to have poor BP control [34,39,40]. Meanwhile, in 2016, another Brazilian study consistently reported that females were more likely to report uncontrolled BP than males [41].

Furthermore, there was a statistically significant association between level of education and the individual’s perspective on more reliance on self-monitoring of blood pressure during the pandemic. Those with higher education seem to be more concerned about their disease management in terms of increased reliance on BP self-monitoring than the other group. In addition, a Canadian study conducted by Gee et al. in 2012 reported that a higher rate of uncontrolled BP was observed among people with low education levels [42].

Moreover, the analysis revealed statistically significant differences in total pandemic impact scores based on age, household income, employment, and marital status. The youngest age group (18–39 years), the lowest income group (B40), unemployed and unmarried individuals had significantly higher pandemic impact scores. This infers that those individuals were more prone to be affected negatively by the pandemic and consequently had more significant challenges to maintain proper hypertension control under this strain. In relation, a recent Singapore study found that being younger, male, and having a lower educational level were all associated with untreated hypertension [43]. However, some studies have suggested that old age is considered as a barrier to optimal BP control and a strong predictor of uncontrolled HTN according to reported data from Singapore and Sweden, respectively [43,44]. In addition, the evidence from an Iraqi study did not support the significant impact of education and employment on the control of BP among hypertensive patients [34].

According to a Nigerian study conducted in 2014, the financial constraints showed an adverse impact on medication adherence, leading to the sub-optimal achievement of BP control [45]. Overall, socio-economically under-privileged people were more likely to show a strong pattern toward the greater risk of having sub-optimal BP level despite treatment [42]. This might underpin the need for customised interventions to target BP control improvement among those individuals with particular socio-economic challenges. Overall, identification of these patients who might have increased challenges with their disease management during the pandemic might suggest a role for relevant health authorities in executing appropriate public health measures and policies to ensure the maintenance of good hypertension management during the ongoing pandemic.

### 4.7. Study Limitations

This study has some limitations, including a relatively small sample (despite repeated and frequent reminders and an extended period of data collection); this could have potentially affected the generalizability of the results to a wider hypertensive population in Malaysia. Moreover, the cross-sectional study design did not allow for assessing the causal relationship. Data could only be collected via an online survey as this was the only probable safe method of data collection amidst the COVID-19-related-restrictions. Additionally, as the survey link was prepared on Google Forms and disseminated through social media, the elderly and those participants without internet access or without social media or Google Forms’ knowledge could not have participated in the study. Furthermore, convenience sampling might have limited the possibility of the sample being representative and predisposed this study to a potential selection bias [46]. Finally, the self-administered questionnaire could have led to potential recall bias [47].

### 4.8. Practical Implications and Future Research

The protracted and unprecedented nature of the current pandemic and the extended challenges warrant an urgent need to optimise care for patients with chronic diseases, such as hypertension. Identifying those patients’ groups at increased risk of developing adverse pandemic impact would help to tailor initiatives, such as online educational programs and innovative tools to assist them in maintaining optimal disease management. Future research should concentrate on testing these findings on a large scale and include a follow-up period to enhance our understanding of how these perceptions and challenges vary over time.

## 5. Conclusions

Most participants reported relatively controlled blood pressure and good levels of health literacy and adherence amid the pandemic. To a moderate extent, study participants perceived that the pandemic had a negative effect on hypertension management. The perceived negative impact of the pandemic was attributed to several socio-demographic factors, such as age, household income, employment, and marital status. These findings may help in identifying the patient groups at higher risk of the pandemic impact to be targeted by relevant initiatives.

## Figures and Tables

**Table 1:** Demographics of study participants and their BP control.

	Frequency	Percentage
**Age Groups**		
18–39	27	18.8
40–59	99	68.8
60 or more	18	12.5
**Gender**		
Male	70	48.6
Female	74	51.4
**Ethnicity**		
Malay	141	97.9
Chinese	3	2.1
**Marital status**		
Unmarried (single/widowed/divorced)	21	14.6
Married	123	85.4
**Level of education**		
Pre-college education	37	25.7
College/University	107	74.3
**Employment status**		
Unemployed/retired	51	35.4
Employed/Self-employed	93	64.6
**Monthly household income**		
Less than RM 4850 (B40 group)	53	36.8
RM 4850–RM 10,959 (M40 group)	64	44.4
More than RM 10,959 (T20 group)	27	18.8
**Location of residence**		
Urban	121	84.0
Rural	23	16.0
**Your average blood pressure reading in the past 7 days**		
Systolic blood pressure <140 mmHg and diastolic blood pressure <90 mmHg	111	77.1
Systolic blood pressure of at least 140 mmHg or diastolic blood pressure of at least 90 mmHg	33	22.9
Total	144	100.0

**Table 2:** Overall responses to health literacy items.

	Frequency	Percentage
**Reading of systolic blood pressure of at least 140 mmHg OR diastolic blood pressure of at least 90 mmHg indicates UNCONTROLLED blood pressure**		
No, not sure	37	25.7
Yes	107	74.3
**Hypertension is a leading risk factor for cardiovascular diseases.**		
No, not sure	14	9.7
Yes	130	90.3
**The treatment of hypertension among patients is a lifelong treatment.**		
No, not sure	29	20.1
Yes	115	79.9
**Reducing the amount of salts intake in diet can reduce blood pressure level.**		
No, not sure	32	22.2
Yes	112	77.8
**Once a person is diagnosed with hypertension, usually he or she will get cured within few weeks after receiving treatment.**		
Yes, not sure	50	34.7
No	94	65.3
Total	144	100.0

**Table 3:** Overall responses to adherence items.

	Frequency	Percentage
**How often do you forget to take your hypertension medicine?**		
Often/very often	11	7.6
Sometimes	28	19.4
Rarely/never	105	72.9
**How often do you decide NOT to take your hypertension medicine?**		
Often/very often	17	11.8
Sometimes	14	9.7
Rarely/never	113	78.5
**How often do you eat food rich in salts?**		
Often/very often	50	34.7
Sometimes	57	39.6
Rarely/never	37	25.7
**How often do you eat fast food?**		
Often/very often	16	11.1
Sometimes	59	41.0
Rarely/never	69	47.9
**How often do you miss scheduled appointments with doctor?**		
Often/very often	11	7.6
Sometimes	17	11.8
Rarely/never	116	80.6
**How often do you forget to get prescriptions filled? (Prescription is an official document written by a doctor for the purpose of patient’s medicine supply)**		
Often/very often	9	6.3
Sometimes	12	8.3
Rarely/never	123	85.4
**How often do you skip taking your hypertension pills intentionally when you feel better?**		
Often/very often	14	9.7
Sometimes	14	9.7
Rarely/never	116	80.6
**How often do you miss taking your hypertension pills when you get sick?**		
Often/very often	11	7.6
Sometimes	13	9.0
Rarely/never	120	83.3
Total	144	100.0

**Table 4:** Responses to items related to items assessing the pandemic impact on hypertension control.

	Frequency	Percentage
**During this pandemic, my blood pressure level becomes worse as compared to before.**		
Disagree/Strongly disagree	93	64.6
Neutral	37	25.7
Agree/Strongly agree	14	9.7
**During this pandemic, I rely more on self-monitoring of blood pressure as compared to before.**		
Agree/Strongly agree	66	45.8
Neutral	48	33.3
Disagree/Strongly disagree	30	20.8
**During this pandemic, I have the fear of going out to get my medications supply due to the risk of COVID-19 infection as compared to before.**		
Disagree/Strongly disagree	81	56.3
Neutral	31	21.5
Agree/Strongly agree	32	22.2
**During this pandemic, my hypertension treatment plan becomes more complex as compared to before.**		
Disagree/Strongly disagree	95	66.0
Neutral	33	22.9
Agree/Strongly agree	16	11.1
**I always practice a healthy diet during this pandemic.**		
Agree/Strongly agree	75	52.1
Neutral	55	38.2
Disagree/Strongly disagree	14	9.7
**Due to this pandemic, I encounter more life-stresses as compared to before.**		
Disagree/Strongly disagree	75	52.1
Neutral	39	27.1
Agree/Strongly agree	30	20.8
**During this pandemic, I seldom attend appointment with doctors as what had been scheduled.**		
Disagree/Strongly disagree	94	65.3
Neutral	30	20.8
Agree/Strongly agree	20	13.9
**I comply with taking the prescribed medications as scheduled during this pandemic.**		
Agree/Strongly agree	106	73.6
Neutral	25	17.4
Disagree/Strongly disagree	13	9.0
Total	144	100.0
